# Clinical curative effect of B-ultrasound-guided minimally invasive percutaneous nephrolithotomy and its influences on stone clearance rate in patients with complex kidney stones

**DOI:** 10.1097/MD.0000000000050729

**Published:** 2026-10-16

**Authors:** Xinjin Gu

**Affiliations:** a Department of Urology, Affiliated People’s Hospital of Ningbo University, Ningbo City, Zhejiang Province, China.

**Keywords:** B-ultrasound guidance, complex kidney stone, minimally invasive, percutaneous nephrolithotomy, stone clearance rate

## Abstract

This study compares short-term perioperative and early postoperative outcomes of B-ultrasound-guided minimally invasive percutaneous nephrolithotomy (MPCNL) and retroperitoneal laparoscopic pyelolithotomy (RLP) in patients with complex kidney stones. This single-center retrospective observational cohort included 120 eligible patients treated between December 2020 and December 2023. Patients were classified according to the treatment actually received (RLP, n = 60; B-ultrasound-guided MPCNL, n = 60); no random allocation was performed. Outcomes included perioperative indicators, 24-hour visual analog scale pain score, 48-hour inflammatory and renal biomarkers, early ultrasound-defined stone clearance at postoperative day 1, and in-hospital complications. Operation time, intraoperative blood loss, and length of hospital stay were lower in the MPCNL group than in the RLP group (nominal *P* < .05). Early ultrasound-defined stone clearance was 91.67% (55/60) versus 78.33% (47/60), corresponding to a risk difference of 13.3 percentage points (95% confidence interval [CI] = 0.2–25.6), a risk ratio of 1.17 (95% CI = 1.00–1.36), and a Pearson chi-square *P* = .041. This comparison did not meet a conservative Bonferroni-adjusted threshold of 0.0042 for 12 reported endpoints. At 24 hours, visual analog scale scores were lower in the MPCNL group, and at 48 hours, inflammatory and renal biomarkers were lower (nominal *P* < .05). Complications occurred in 2/60 (3.33%) versus 8/60 (13.33%); because of sparse events, Fisher exact test was used (*P* = .095; risk ratio = 0.25, 95% CI = 0.06–1.13). B-ultrasound-guided MPCNL was associated with more favorable short-term perioperative and biomarker outcomes and a nominally higher early ultrasound-defined clearance rate than RLP. However, the retrospective nonrandomized design, incomplete baseline adjustment, short follow-up, outcome-measurement limitations, and multiple comparisons preclude causal or long-term effectiveness claims. These findings should be considered hypothesis-generating and require confirmation in adequately powered prospective comparative studies.

## 1. Introduction

Kidney stones (KS) form when minerals in urine become supersaturated and crystallize, resulting in solid mineral and salt deposits in the kidneys.^[1]^ Complex KS refer to those that are more difficult to treat and may involve multiple factors, severe symptoms, or recurrence. The formation mechanism of complex KS is complex and may be influenced by various factors. These factors may act alone or in synergy, causing certain components in the urine to become supersaturated and subsequently forming stones in the kidneys.^[2]^ The morphological structure of complex KS is also more complex, possibly presenting as antler-shaped, cast-like, or branched forms, etc. This complex morphological structure not only increases the volume and weight of the stones but also may affect the excretion of urine, leading to renal function impairment or urinary tract obstruction and other serious consequences.^[3]^ Currently, there are many treatment methods for complex KS in clinical practice, and their treatment has always been a key research content in the field of urology. Traditional treatment methods such as extracorporeal shock wave lithotripsy and retroperitoneal laparoscopic pyelolithotomy (RLP) have certain limitations and cannot meet the treatment needs of all patients.^[2,4]^ In recent years, with the continuous advancement of medical technology, B-ultrasound-guided minimally invasive percutaneous nephrolithotomy (MPCNL) as a new treatment method has gradually attracted attention. This technique combines the advantages of B-ultrasound real-time guidance and minimally invasive technology, providing new treatment options for patients with KS.^[5]^ This study analyzed the clinical efficacy and stone clearance rate of patients with complex KS treated by 2 different surgical methods, RLP and B-ultrasound-guided MPCNL, to evaluate the clinical application value of this minimally invasive technique.

## 2. Materials and methods

### 2.1. General information

This was a single-center retrospective observational cohort study of patients with complex KS treated at the Affiliated People’s Hospital of Ningbo University between December 2020 and December 2023. A total of 120 eligible patients were included. The study was approved by the institutional ethics committee. The analysis was based on existing clinical records; therefore, treatment assignment was not randomized.

Inclusion criteria were: complex KS meeting the diagnostic criteria cited in Türk et al^[6]^ and documented on imaging; for the present analysis, complex stones included multiple renal calculi and partial or complete staghorn/branched (cast-like) configurations; bilateral renal function considered adequate for surgery; no surgical contraindications; and documented consent for clinical treatment. Exclusion criteria were: nephritis, perinephric infection, or other major urinary tract disease; previous gastrointestinal surgery or bleeding disorder; previous kidney stone surgery; renal or ureteral malformation; severe organ disease or malignancy; and severe psychiatric disorder or inability to communicate. Patients were classified according to the operation documented in the medical record: RLP (control group, n = 60) or B-ultrasound-guided MPCNL (study group, n = 60). Baseline characteristics in the analytic manuscript dataset were age, sex, and stone side: control group, 33 men/27 women, mean age 53.52 ± 6.42 years, 34 left/26 right; MPCNL group, 35 men/25 women, mean age 53.65 ± 6.25 years, 36 left/24 right. No statistically significant differences were detected for these limited variables (*P* > .05).

Patients meeting the eligibility criteria were identified from the available clinical records during the predefined study period. No randomization, prospective allocation, or matching procedure was performed. The choice of surgical procedure was made during routine clinical care before inclusion in this retrospective analysis. Patients were subsequently classified according to the treatment actually documented in their medical records as RLP (n = 60) or B-ultrasound-guided MPCNL (n = 60).

B-ultrasound-guided MPCNL was therefore not assigned according to a study-specific research protocol. In routine clinical practice, procedural selection could have been influenced by preoperative imaging findings and stone anatomy, the patient’s general clinical condition and preferences, and the treating surgeon’s judgment regarding procedural feasibility. Because these factors were involved in clinical decision-making rather than controlled by the study investigators, selection bias and confounding by indication are possible.

The baseline variables sufficiently complete for analysis were age, sex, and stone side. Other potentially important determinants of both procedural selection and clinical outcomes, including total stone burden or maximum stone diameter, detailed stone location, computed tomography (CT) attenuation, BMI, ASA class or Charlson Comorbidity Index, baseline eGFR, hydronephrosis, and preoperative urine culture status, were not sufficiently complete in the available dataset for reliable retrospective adjustment. Therefore, no multivariable-adjusted or propensity-score analysis was performed, and similarity in the available baseline variables should not be interpreted as evidence that the 2 treatment groups were fully comparable.

### 2.2. Methods

Before the operation, both groups of patients needed to undergo a comprehensive physical examination, including blood tests, urine tests, and renal function assessment, to determine the patient’s physical condition and the specific situation of the stones. At the same time, based on the size, location, and number of the stones, a detailed surgical plan was formulated. In addition, imaging examinations were also conducted, such as X-ray examinations, B-ultrasound examinations, urography, and urinary system CT examinations, to determine the size, quantity, location, and shape of the stones, providing precise positioning for the surgery.

The control group patients were treated with RLP. The patients would undergo tracheal intubation under general anesthesia and have a urinary catheter inserted, preparing for surgery. During the operation, the patients would assume a lateral recumbent position on the affected side. Then, a surgical channel would be established through the retroperitoneal route. Usually, a cannula would be inserted 2 cm below the 12th rib margin on the posterior axillary line, 1 cm below the 12th rib margin on the anterior axillary line, and 2 cm above the iliac spine on the midaxillary line, respectively. Through the puncture technique, the laparoscope and related instruments would be introduced into the abdominal cavity, opening the Gerota fascia and pushing the peritoneum aside, finding the renal pelvis and cutting it, and directly observing the renal pelvis under the laparoscope to find and remove the stones. For larger or cast-like stones, it may be necessary to first insert a nephroscope through the renal pelvis incision, using the ballistic lithotripsy technique to break the stones, and then remove them one by one. During the process of removing the stones, the integrity of the stones must be ensured, and any residual small stones should be carefully checked. For cast-like stones or those that are difficult to remove, the doctor may insert a nephroscope through the renal pelvis incision first, perform ballistic lithotripsy, and then remove the stones. The renal pelvis and urinary system would be rinsed with normal saline to ensure that the stone fragments and debris are completely flushed out. After removing all the stones, an F5 double-J tube would be placed internally, the renal pelvis incision would be sutured with absorbable sutures, and the puncture site would be closed to ensure a secure suture without affecting kidney function. After the renal pelvis incision is sutured, the abdominal cavity would be rinsed again to ensure the cleanliness of the surgical area. In addition, to ensure the cleanliness of the surgical area and prevent postoperative infection, a retroperitoneal drainage tube would be placed, the surgical instruments would be withdrawn, and the surgery would be completed.

The patients in the study group were treated with MPCNL under the guidance of B-ultrasound. Before the start of the surgery, the doctor would use the B-ultrasound equipment to determine the exact location and size of the stones, as well as the specific shape of the kidney. The patients received anesthesia, usually spinal epidural block anesthesia or general anesthesia. Depending on the location of the stones, the patients may need to adopt specific positions, such as prone or lateral, to facilitate the doctor’s puncture operation. Under the real-time guidance of B-ultrasound, a special puncture needle was used to precisely insert into the kidney and reach the location of the stones. After successful puncture, a channel, namely the fistula, was established using the puncture needle. Through the fistula, the stones were removed or broken into small pieces using stone removal forceps or stone-breaking equipment, facilitating their excretion from the body. During this process, the doctor would closely monitor the patient’s vital signs and the surgical situation to ensure the safe conduct of the surgery. After the surgery, the puncture needle was withdrawn, and bleeding from the fistula was stopped and disinfected. The patients were required to maintain an appropriate position after the surgery to facilitate drainage and prevent urine leakage.

Both groups of patients needed to undergo close observation after the surgery, including vital signs, urine conditions, wound recovery, etc. At the same time, according to the doctor’s instructions, antibiotics were used to prevent infection, and appropriate rehabilitation training and dietary adjustments were carried out.

### 2.3. Observation indicators

#### 2.3.1. Comparison of perioperative related indicators between the 2 groups

The operation time, intraoperative blood loss, and hospital stay were recorded. Early stone clearance was assessed by B-ultrasonography on postoperative day 1. The prespecified operational criterion used in the original dataset classified patients as “cleared” when no residual stone was visualized or when the largest residual fragment was <2 mm. This endpoint is termed early ultrasound-defined stone clearance rather than true stone-free status, because ultrasonography is operator-dependent and less sensitive than CT for small residual fragments, and fragments <2 mm may remain clinically relevant during longer follow-up.

#### 2.3.2. Comparison of pain degree between the 2 groups

The pain condition was evaluated using the visual analog scale (VAS) before the operation and 24 hours after the operation.^[7]^ The total score was 0 to 10 points, with a higher score indicating more obvious pain.

#### 2.3.3. Comparison of inflammatory indicators between the 2 groups

Five milliliters of morning peripheral venous blood were drawn from the 2 groups of patients before and 48 hours after the operation, mixed with heparin to make anticoagulated blood, and left to stand at room temperature for 30 minutes before centrifugation to obtain the upper layer serum. One part of it was stored at −75°C for long-term refrigeration. The serum levels of high-sensitivity C-reactive protein (hs-CRP) were measured using a domestic Prolan special protein analyzer according to the operation standards and recorded. The levels of interleukin-6 (IL-6) and tumor necrosis factor (TNF-α) in the patients were detected using an enzyme-linked immunosorbent assay kit.

#### 2.3.4. Comparison of renal function indicators between the 2 groups

Before and 48 hours after the operation, another sample of serum was taken, and the levels of blood urea nitrogen (BUN), serum creatinine (Scr), and neutrophil gelatinase-associated lipocalin (NGAL) were detected using the enzyme-linked immunosorbent assay kit from R&D Systems in the UK.

#### 2.3.5. Comparison of complication rates between the 2 groups

Postoperative imaging assessment was performed by radiology personnel who were not involved in the operation; formal blinding of image readers to the treatment received could not be verified from the currently available records. Patients, surgeons, VAS assessment, and perioperative data collection were not blinded. Postoperative bleeding, urinary leakage, and infection were recorded during the index treatment period. Complications were not prospectively graded using the Clavien-Dindo system, and the archived manuscript dataset did not contain sufficiently detailed treatment-based severity information to reconstruct Clavien-Dindo grades reliably. Future studies should prospectively define and grade complications using a standardized system.

### 2.4. Statistical methods

Analyses were performed with Statistical Package for the Social Sciences 25.0. Continuous variables were summarized as mean ± standard deviation and compared between groups using unadjusted independent-samples *t* tests as in the original analysis. Binary outcomes were summarized as n (%) with risk differences and risk ratios with 95% confidence intervals. Pearson chi-square test was used when cell counts were adequate; Fisher exact test was used for sparse events, including the complication comparison. All tests were two-sided. Because 12 distinct endpoints were reported (operation time, blood loss, length of stay, early stone clearance, VAS, hs-CRP, IL-6, TNF-alpha, Scr, BUN, NGAL, and complications), nominal *P* values are interpreted as exploratory; a conservative Bonferroni reference threshold is 0.05/12 = 0.0042. No a priori sample-size or power calculation was performed because the retrospective sample comprised the eligible records available during the study period. Precision is therefore emphasized through 95% confidence intervals where calculable. No multivariable or propensity-score adjustment was possible from the manuscript dataset because important baseline covariates were unavailable. Consequently, adjusted causal treatment effects should not be inferred.

## 3. Results

### 3.1. Comparison of perioperative related indicators between the 2 groups

The perioperative related indicators of the control group were all higher than those of the study group (*P* < .05); the stone clearance rate of the study group was higher than that of the control group (*P* < .05), as shown in Table 1.

**Table 1:** Comparison of perioperative indicators between the 2 groups (±s).

Outcome	MPCNL (n = 60)	RLP (n = 60)	Effect estimate (95% CI)	Hedges’ *g*	Nominal *P*	Holm-adjusted *P*
Operative time (min)	72.68 ± 12.54	85.79 ± 12.72	−13.11 (−17.68 to −8.54)	−1.03	<.001	<.001
Intraoperative blood loss (mL)	55.64 ± 4.37	78.33 ± 7.42	−22.69 (−24.90 to −20.48)	−3.70	<.001	<.001
Length of hospital stay (d)	11.73 ± 2.45	15.62 ± 3.24	−3.89 (−4.93 to −2.85)	−1.35	<.001	<.001
Initial stone-free rate, n (%)	55 (91.67)	47 (78.33)	1.17 (1.00 to 1.36); RD = 13.3% (0.8 to 25.9%)	–	.041	–

CI = confidence interval, MPCNL = minimally invasive percutaneous nephrolithotomy, RLP = retroperitoneal laparoscopic pyelolithotomy.

### 3.2. Comparison of pain severity between the 2 groups

The VAS score of the control group at 24 hours after surgery was higher than that of the study group (*P* < .05), as shown in Table 2.

**Table 2:** Comparison of pain severity between the 2 groups ([±s], points).

Time point	MPCNL (n = 60)	RLP (n = 60)	MD (95% CI)	Hedges’ *g*	Nominal *P*	Holm-adjusted *P*
Preoperative	4.72 ± 0.49	4.65 ± 0.54	0.07 (−0.12 to 0.26)	0.13	.459	–
24-h postoperative	2.32 ± 0.45*	2.91 ± 0.55*	−0.59 (−0.77 to −0.41)	−1.17	<.001	<.001

Compared with the preoperative conditions of the same group.

CI = confidence interval, MPCNL = minimally invasive percutaneous nephrolithotomy, RLP = retroperitoneal laparoscopic pyelolithotomy.

* *P* < .05.

### 3.3. Comparison of inflammatory indicators between the 2 groups

The levels of hs-CRP, IL-6, and TNF-α in the control group at 48 hours after surgery were all higher than those in the study group (*P* < .05), as shown in Table 3.

**Table 3:** Comparison of inflammatory indicators between the 2 groups (±s).

Marker	Time point	MPCNL (n = 60)	RLP (n = 60)	MD (95% CI)	Hedges’ *g*	Nominal *P*	Holm-adjusted *P*
hs-CRP (mg/L)	Preoperative	5.68 ± 0.74	5.59 ± 0.81	0.09 (−0.19 to 0.37)	0.12	.526	–
hs-CRP (mg/L)	48 h	8.90 ± 0.88	10.87 ± 1.23	−1.97 (−2.36 to −1.58)	−1.83	<.001	<.001
IL-6 (pg/mL)	Preoperative	23.54 ± 4.38	23.47 ± 4.40	0.07 (−1.52 to 1.66)	0.02	.931	–
IL-6 (pg/mL)	48 h	30.63 ± 6.12	40.87 ± 6.64	−10.24 (−12.55 to −7.93)	−1.59	<.001	<.001
TNF-α (μg/L)	Preoperative	4.60 ± 0.72	4.55 ± 0.74	0.05 (−0.21 to 0.31)	0.07	.708	–
TNF-α (μg/L)	48 h	9.64 ± 1.36	11.51 ± 1.69	−1.87 (−2.42 to −1.32)	−1.21	<.001	<.001

CI = confidence interval, hs-CRP = high-sensitivity C-reactive protein, IL-6 = interleukin-6, MPCNL = minimally invasive percutaneous nephrolithotomy, RLP = retroperitoneal laparoscopic pyelolithotomy, TNF-α = tumor necrosis factor-α.

### 3.4. Comparison of renal function indicators between the 2 groups

The levels of Scr, BUN, and NGAL in the control group at 48 hours after surgery were all higher than those in the study group (*P* < .05), as shown in Table 4.

**Table 4:** Comparison of renal function indicators between the 2 groups (±s).

Marker	Time point	MPCNL (n = 60)	RLP (n = 60)	MD (95% CI)	Hedges’ *g*	Nominal *P*	Holm-adjusted *P*
SCr (μmol/L)	Preoperative	58.40 ± 7.37	59.32 ± 7.85	−0.92 (−3.67 to 1.83)	−0.12	.509	–
SCr (μmol/L)	48 h	71.44 ± 7.95	82.61 ± 8.32	−11.17 (−14.11 to −8.23)	−1.36	<.001	<.001
BUN (reported units)	Preoperative	5.69 ± 0.54	5.75 ± 0.57	−0.06 (−0.26 to 0.14)	−0.11	.555	–
BUN (reported units)	48 h	6.65 ± 0.74	7.34 ± 0.81	−0.69 (−0.97 to −0.41)	−0.88	<.001	<.001
NGAL (μg/L)	Preoperative	3.50 ± 0.49	3.45 ± 0.53	0.05 (−0.13 to 0.23)	0.10	.593	–
NGAL (μg/L)	48 h	4.60 ± 0.79	5.37 ± 0.85	−0.77 (−1.07 to −0.47)	−0.93	<.001	<.001

BUN = blood urea nitrogen, CI = confidence interval, MPCNL = minimally invasive percutaneous nephrolithotomy, NGAL = neutrophil gelatinase-associated lipocalin, RLP = retroperitoneal laparoscopic pyelolithotomy, SCr = serum creatinine.

### 3.5. Comparison of complication rates between the 2 groups

After the operation, the complication rate of the study group was lower than that of the control group (*P* < .05), as shown in Table 5.

**Table 5:** Comparison of complication incidence rates between the 2 groups (n [%]).

Outcome	MPCNL (n = 60)	RLP (n = 60)	Effect estimate	95% CI	χ^2^	*P* value	Holm-adjusted *P*
Postoperative bleeding	0 (0.00)	2 (3.33)	Descriptive	–	–	–	–
Infection	1 (1.67)	3 (5.00)	Descriptive	–	–	–	–
Urinary leakage/incontinence	1 (1.67)	3 (5.00)	Descriptive	–	–	–	–
Any complication	2 (3.33)	8 (13.33)	RR = 0.25; RD = −10.0%	RR = 0.06 to 1.13; RD = −19.7% to −0.3%	3.927	.048	.048

CI = confidence interval, MPCNL = minimally invasive percutaneous nephrolithotomy, RLP = retroperitoneal laparoscopic pyelolithotomy.

## 4. Discussion

Complex renal calculi include multiple renal calculi, partial arc-shaped renal calculi, and complete arc-shaped renal calculi. Their treatment has always been a challenge in the field of urology.^[3]^ Traditional surgical treatment has many shortcomings. Therefore, finding a safe, effective, and minimally invasive treatment method has become the focus of clinical research. In recent years, with the continuous development of minimally invasive technology, MPCNL technology, as a new type of minimally invasive treatment method, has gradually attracted widespread attention.^[8]^ This study aims to deeply explore the clinical efficacy and stone clearance rate of MPCNL treatment for patients with complex renal calculi in order to provide more effective treatment options for clinical practice.

In this study, the perioperative indicators such as operation time, intraoperative blood loss, and hospital stay of patients in the B-ultrasound-guided MPCNL treatment group were lower than those in the RLP treatment group. This is because B-ultrasound, as a noninvasive and real-time imaging technology, can clearly display the structure of the kidney and the location of the stones, providing accurate positioning information for the surgeons.^[9]^ The MPCNL technology uses B-ultrasound guidance, making the surgical operation more precise and controllable. In contrast, RLP surgery requires a larger surgical incision and extensive dissection of the kidney during the operation, making the operation relatively complex, so the operation time is relatively longer.^[9]^ MPCNL establishes a channel through percutaneous nephroscopy, avoiding the extensive dissection and exposure of the kidney during RLP surgery. This minimally invasive approach significantly reduces surgical trauma, protects the integrity of the kidney and surrounding tissues, and reduces the risk of intraoperative bleeding.^[9]^ At the same time, due to the small surgical trauma, postoperative recovery is also faster, allowing patients to return to normal life and work earlier. MPCNL technology also adopts advanced lithotripsy equipment, with higher lithotripsy efficiency, and can more thoroughly remove stones, reducing the possibility of stone residue, which also provides a possibility for reducing the operation time.^[10]^ At the same time, MPCNL technology has less impact on patients, with lower postoperative pain and inflammation response. This enables patients to get out of bed earlier, promoting the speed of postoperative recovery. Therefore, the hospital stay is also shortened, further reducing medical costs and social burdens.^[11]^ The results of this study show that the stone clearance rate of the study group is higher than that of the control group, and the pain score at 24 hours after surgery is lower than that of the control group. This is because MPCNL technology uses real-time guidance from B-ultrasound to precisely locate the position and size of the stones. This precise positioning enables surgeons to perform lithotripsy and stone removal more accurately, thereby increasing the stone clearance rate. In contrast, although RLP surgery can directly expose and remove the stones, due to its large surgical trauma and complex operation, it may have certain limitations in the treatment of complex renal calculi, and it may be difficult to completely clear some stones in special positions. MPCNL technology adopts a minimally invasive approach, avoiding the large incision and extensive tissue dissection during RLP surgery. Due to the small surgical trauma, the postoperative pain is also relatively lower. In addition, the minimally invasive approach of MPCNL significantly reduces the damage to the kidney and surrounding tissues during the surgery, thereby reducing the degree of postoperative inflammation.^[12]^ In contrast, RLP surgery often causes more severe inflammatory reactions due to the large-scale surgical operation and tissue damage. Laparoscopic surgery requires the use of carbon dioxide to establish an intra-abdominal pneumoperitoneum for surgical operations. Although carbon dioxide plays an important role in the surgery, it may also have a certain stimulating effect on tissues, triggering inflammatory reactions. Moreover, the accumulation and metabolism of carbon dioxide in the body may also affect the inflammatory response. In this study, the levels of inflammatory markers hs-CRP, IL-6, and TNF-α in the MPCNL group were lower than those in the RLP surgery group at 48 hours after surgery, which precisely confirmed this point. The research results also revealed that the levels of renal function indicators such as Scr, BUN, and NGAL, as well as the incidence of postoperative complications, in the MPCNL group were lower than those in the control group at 48 hours after surgery. The presence of stones may cause problems such as urinary tract obstruction and hydronephrosis, which in turn affect renal function.^[13]^ The MPCNL technique can more accurately locate and remove stones, thereby reducing the obstruction and damage of stones to the kidneys. At the same time, the MPCNL technique has a faster postoperative recovery, allowing patients to get out of bed earlier and undergo rehabilitation training. This helps to promote metabolism and blood circulation, reduce the accumulation and release of inflammatory substances, and thereby help protect renal function. In addition, the high precision, visualization, minimally invasive nature, rapid recovery, short operation time, and high stone clearance rate characterize B-ultrasound-guided MPCNL surgery. These factors together make MPCNL a safe and effective option for the treatment of complex KS.

This study has several important limitations. First, it is a retrospective, nonrandomized observational study. Because treatment was determined clinically, selection bias and confounding by indication are unavoidable concerns. Patients with different stone burden, anatomy, comorbidity, renal function, infection status, or operative risk may preferentially receive one procedure, and these same factors may influence clearance, recovery, and complications. Second, baseline characterization was incomplete. Only age, sex, and stone side were available in the analytic manuscript dataset, whereas stone burden/size, calyceal or pelvic location, CT density, BMI, ASA/Charlson comorbidity, baseline eGFR, hydronephrosis, and urine culture status are clinically relevant. A nonsignificant comparison of only 3 baseline variables cannot demonstrate exchangeability between treatment groups. Because these variables were unavailable, no valid adjusted or propensity-based analysis could be performed. This materially limits causal interpretation and may explain part of the observed group differences. Third, follow-up was limited to 24 to 48 hours for pain and laboratory outcomes, and stone clearance was assessed by ultrasonography on postoperative day 1. Ultrasonography is operator-dependent and may miss small residual fragments compared with CT. Moreover, the operational threshold that counted residual fragments <2 mm as “cleared” should not be equated with true stone-free status. Contemporary EAU follow-up guidance defines stone-free status as no residual fragments on postoperative imaging and recognizes that residual fragments require follow-up; even fragments ≤4 mm show non-negligible rates of progression and later intervention. Thus, the present study cannot address recurrence, delayed passage or growth of residual fragments, re-intervention, readmission, or sustained renal recovery. Future studies should include standardized imaging at clinically meaningful intervals (e.g., 4–12 weeks and longer-term follow-up), document residual-fragment size and location, and collect recurrence and re-intervention outcomes. Finally, the term “complex kidney stones” encompasses heterogeneous anatomy and burden. Although the revised methods provides an operational description based on the available imaging categories, the current dataset does not permit robust stratification by staghorn extent, number, location, size, density, or validated stone-complexity scores. Treatment effects may differ substantially across these subgroups. Future studies should use standardized stone-burden measures and validated complexity scores and should report stratified or interaction analyses. Finally, this was a single-center study, which may limit generalizability to centers with different expertise, equipment, and perioperative pathways.

In this retrospective single-center cohort, B-ultrasound-guided MPCNL was associated with shorter operation time, less intraoperative blood loss, shorter hospitalization, lower postoperative pain and biomarker levels, and a nominally higher early ultrasound-defined stone clearance rate than RLP. However, because treatment selection was nonrandomized and important baseline characteristics related to stone complexity, comorbidity, renal function, and infection status were unavailable for adjustment, the observed differences cannot be interpreted as causal treatment effects. These findings should therefore be considered exploratory and require confirmation in prospective studies with standardized assessment of stone complexity, adequate control of confounding, and longer-term follow-up.

## Author contributions

**Conceptualization:** Xinjin Gu.

**Data curation:** Xinjin Gu.

**Formal analysis:** Xinjin Gu.

**Funding acquisition:** Xinjin Gu.

**Investigation:** Xinjin Gu.

**Writing – original draft:** Xinjin Gu.

**Writing – review & editing:** Xinjin Gu.
